# Synthesis and Biological Evaluation of New Cholinesterase Inhibitors for Alzheimer’s Disease

**DOI:** 10.3390/molecules23082033

**Published:** 2018-08-14

**Authors:** Weiam Hussein, Begüm Nurpelin Sağlık, Serkan Levent, Büşra Korkut, Sinem Ilgın, Yusuf Özkay, Zafer Asım Kaplancıklı

**Affiliations:** 1 Department of Pharmaceutical Chemistry, Faculty of Pharmacy, Aden University, 6075 Aden, Yemen; white.wf.rose@gmail.com; 2 Department of Pharmaceutical Chemistry, Faculty of Pharmacy, Anadolu University, 26470 Eskişehir, Turkey; bnsaglik@anadolu.edu.tr (B.N.S.); serkanlevent@anadolu.edu.tr (S.L.); zakaplan@anadolu.edu.tr (Z.A.K.); 3 Doping and Narcotic Compounds Analysis Laboratory, Faculty of Pharmacy, Anadolu University, 26470 Eskişehir, Turkey; 4 Department of Pharmaceutical Toxicology, Faculty of Pharmacy, Anadolu University, 26470 Eskişehir, Turkey; busrakorkut@anadolu.edu.tr (B.K.); silgin@anadolu.edu.tr (S.I.)

**Keywords:** Alzheimer’s disease, acetylcholinesterase, butyrylcholinesterase, 9-aminoacridine, dithiocarbamate salts, docking study

## Abstract

Alzheimer’s disease (AD) is a neurodegenerative disorder mostly influencing the elderly, and causes death due to dementia. The main pathogenic feature connected with the progression of this multifactorial disease is the weakening of the cholinergic system in the brain. Cholinesterase (ChE) inhibitors are recognized as one of the choices in the treatment of AD. The inhibition of acetylcholinesterase (AChE) and butyrylcholinesterase (BChE) were approved as a therapeutic strategy to reduce the symptoms of AD and prevent its progression. The capacity of BChE is not completely known yet; rather, it is accepted to assume a part in a few disorders such as AD. Thus, BChE inhibitors may have a greater role for the treatment of AD in the future. In the present study, 2-(9-acridinylamino)-2-oxoethyl piperazine/piperidine/morpholinecarbodithioate derivatives were synthesized in order to investigate anticholinesterase activity. Eight derivatives demonstrated a specific and promising action against BChE. Furthermore, compound **4n** showed inhibitory activity against both enzymes. It was found that the active compounds were well tolerated in the cytotoxicity test. Possible interactions between the lead compound, **4n**, and the BChE enzyme were determined through a docking study. The findings obtained within this paper will contribute to the development of new and effective synthetic anti-Alzheimer compounds, and will ideally encourage future screening against AD.

## 1. Introduction

Alzheimer’s disease (AD) is a neurological disorder and is one of the most common causes of dementia in the age group above 60 years, for which there is no radical cure. It is estimated from the world population that more than 35.6 million people are currently living with AD, and this may increase to 65.7 million by 2030, and 115.4 million by 2050 [1]. According to a World Health Organization (WHO) report, more than 50% of people with AD live in the developing world, and it is estimated that this population will be 70% by 2025 [2]. To date, some cases of low levels of acetylcholine (ACh), oxidative anxiety, dyshomeostasis of biometals, and amyloid-β (Aβ) stores were exhibited to be related to AD pathogenesis [3]. In light of these findings, a few speculations were proposed to clarify the progress of AD. The most recognized theory includes the loss of cholinergic neurons in the cerebrum of patients with AD, and a reduced action of choline acetyltransferase, which catalyzes the generation of ACh. Thus, cognitive dysfunction occurs and neurotransmission levels decrease [4]. In AD, although AChE is generally the primary medication target, recent investigations were additionally centered on a scan for BChE inhibitors [5,6,7,8]. One of the most interesting studies showed that, when BChE progresses toward the formation of Aβ plaques, its advanced role involves the conversion of “harmless” plaques to “damaging” plaques [9]. Scientists additionally discovered a relationship between a less active variant of BChE (Ala539Thr) and a lower inclination toward growing AD [10]. Therefore, BChE represents a proper imaging target for early diagnosis and treatment of AD. In this way, new remedial approaches are presently in progress, and exploratory medicine surrounding this approach sped up during the recent decade.

Carbamates are the most generally contemplated class of AChE inhibitors, on which impressive research was performed in connection to AD. Rivastigmine, a dual AChE and BChE inhibitor, is one of the most broadly utilized anticholinesterase agents bearing a carbamate moiety [11,12]. Dithiocarbamates have a great deal of interest in the design of new compounds, which can be obtained via the bioisosteric substitution of a carbamate moiety with a dithiocarbamate moiety. They are critical pharmacophores due to their lipophilicity, which is essential for the transport of drugs to the central nervous system (CNS) through the blood–brain barrier (BBB) [13,14]. Tacrine and donepezil are other anticholinesterase agents that selectively inhibit BChE and AChE, respectively. The use of tacrine is limited as it may cause an increase in the level of serum aminotransferase, which is connected to intense liver harm. On the other hand, donepezil is a more favorable AChE inhibitor since it has some advantages such as non-hepatotoxicity, and the lowest number of side effects when consumed once daily [15]. Thus, several studies on compounds structurally related to tacrine were performed to discover new BChE inhibitors with reduced side effects [16,17,18,19,20,21,22,23]

**Figure 1:**
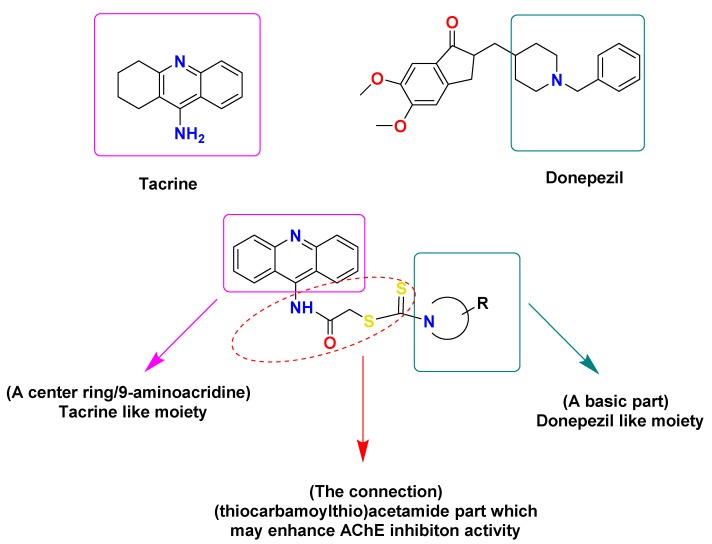
Design strategy of recently synthesized derivatives.

It was reported that anticholinesterase agents ought to feature a central ring, a basic region, and a suitable connection between the central ring and basic region, such as O, CH_2_, CONH, and CONH(CH_2_)n, to satisfy the main structural necessity for active and strong enzyme inhibitors [15]. In addition, the bulky moieties were reported to possess good anti-BChE activity, which may be related to the ability of BChE to ideally bind with bigger substituents, as its structure is composed of a more open core [24]. Furthermore, there are no reported data on 9-aminoacridine and its acetylated analogs as anticholinesterase inhibitors until this study. Based on this empirical knowledge, we aimed to improve BChE inhibitory activity via the design of new compounds bearing the following structural properties: 9-aminoacridine as the central ring, heterocyclic rings such as piperazine, piperidine, or morpholine as the basic region, and finally, a (thiocarbamoylthio)acetamide moiety as the connection between the central ring and the basic region (Figure 1). Thus, the aim was to synthesize new hybrids of 9-aminoacridine and dithiocarbamates analogs as anticholinesterase agents.

## 2. Results and Discussion

### 2.1. Chemistry

**Scheme 1:**
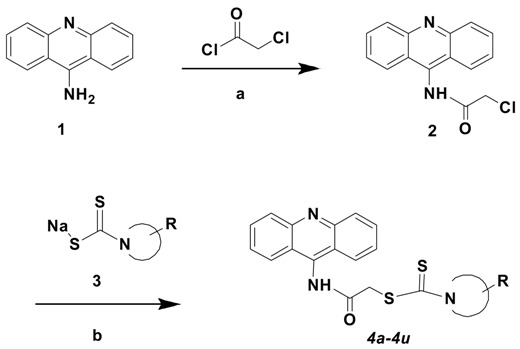
Synthesis of compounds **4a**–**4u**. (**a**): (C_2_H_5_)_3_N/THF/0 °C then rt; (**b**) acetone/reflux.

The newly synthesized derivatives (**4a**–**4u**) were obtained by taking advantage of the three-step protocol as outlined in Scheme 1. The treatment of *N*-(9-acridinyl)-2-chloroacetamide derivatives (**2**) with the appropriate sodium *N*-substituted piperazine/piperidine/morpholine dithiocarbamates (**3**) gave the target compounds with yields of 81.0–97.0%. The structures of all synthesized compounds were elucidated using infrared (IR), high-resolution mass spectrometry (HRMS), ^1^H-NMR, and ^13^C-NMR spectroscopic methods. All compounds indicated logical, analytical, and spectroscopic information strongly correlating with their structures.

### 2.2. Enzymatic Inhibition

The results of biological assays were evaluated to understand the inhibitory potency of the newly synthesized compounds (**4a**–**4u**) against cholinesterase enzymes of different species (AChE from *Electrophorus electricus* and human cells, and BChE from equine and human cells). In order to acquire effective knowledge about structure activity relationship (SAR), compounds **4a**–**4u** were divided into two classes as derivatives containing piperazine (**4a**–**4q**) and morpholine or piperidine (**4r**–**4u**). The assay was performed in two steps. Firstly, compounds **4a**–**4u** were tested at 10^−3^ and 10^−4^ M concentrations. The second step was performed by using 10^−5^–10^−9^ M concentrations of selected compounds that indicated more than 50% inhibitory activity at the initial concentrations. It was clearly observed that similar results were obtained against both species of AChE and BChE. The reason for this similarity was thought to be high degree of homology in the enzymes of the studied species (Supplementary Materials and Table 1).

Our in-depth analysis showed that the incorporation of a 9-aminoacridine moiety into the designed compounds contributed weakly to the AChE inhibitory activity; however, it strongly increased the inhibitory activity toward BChE (Supplementary Materials and Table 1).

Compounds in the first class (**4a**–**4q**) offered inhibitory activity with an IC_50_ ranging from 0.014 to 2.097 µM against BChE. Compounds **4a**, **4b**, **4e**, **4i**, **4m**, **4n**, and **4o** possessing piperazine with 2-dimethylaminoethyl, 3-dimethylaminopropyl, 2-hydroxyethyl, 4-chlorophenyl, 4-benzhydryl, 4-(trifluoromethyl)benzyl, and 4-methylbenzyl respectively, showed more potent BChE inhibitory activity than other derivatives in the series. Furthermore, the most active compound, **4m** (IC_50_: 0.092 µM), exhibited 15.4-fold better BChE inhibition in comparison with the positive control, donepezil (IC_50_: 1.419 µM). In the second class of compounds, the replacement of piperazine with its bioisosters, namely morpholine (**4r**) and piperidine (**4s**, **4t**, and **4u**), did not significantly enhance the activity against either AChE or BChE.

In terms of SAR, it can be concluded that the variation of secondary amines such as piperazine, piperidine, and morpholine does not affect the enzyme inhibitory potency of the final compounds. However, the substituents on these cyclic amines are mainly responsible for the biological activity. Although the structures of the compounds show great similarities, the presence of trifluoromethyl in compound **4n** significantly influenced the activity. The better inhibitory profile of **4n** than aminoacridine (Table 1) also supports this finding. Thus, it can be suggested that the trifluoromethyl group possesses the ability to form interactions in the enzyme’s active site.

It was reported that BChE contains residues which permit bulkier substrates to enter into the active site [25]. Due to the presence of an acridine ring, the synthesized compounds are big molecules; thus, they may have a higher tendency to inhibit BChE compared to AchE. This situation is also reflected in Table 1. At 10^−4^ M concentration, all compounds possessed higher inhibition against BChE**,** indicating that the synthesized compounds had more selectivity toward BChE than AcHE.

### 2.3. Kinetics Characterization of BChE Inhibition

Recognizing the types of enzyme inhibitors is vital; however, much of the drug discovery process focuses instead upon deciding whether one inhibitor is more powerful than another. The two most basic values for evaluating the inhibitor’s power and characteristics are IC_50_ and K_i_ [26].

**Figure 2:**
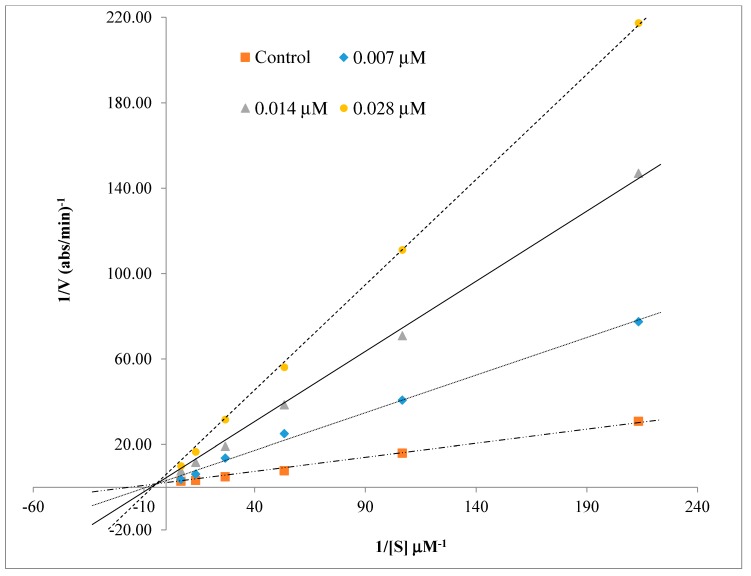
Lineweaver–Burk plot for the inhibition of human butyrylcholinesterase (BChE) by compound **4n** at different concentrations of substrate butyrylthiocholine iodide (BTCI).

To recognize the inhibition type, enzyme kinetics studies were applied using Lineweaver–Burk plots [27]. For this purpose, we selected compound **4n**, which showed the best activity against human BChE. There are four different points on the graphs showing the presence (at concentrations of IC_50_/2, IC_50_, and 2 × IC_50_) and absence (control) of **4n**. As seen in Figure 2, every single straight line intersected in the second quadrant of the coordinate axis, which characterizes a typical mixed inhibition. In addition, a graphical examination of the corresponding Lineweaver–Burk plot shows both increased slopes (decreased V_max_) and intercepts (higher K_m_) at higher inhibitor concentration. This pattern also indicates a mixed-type inhibition.

**Figure 3:**
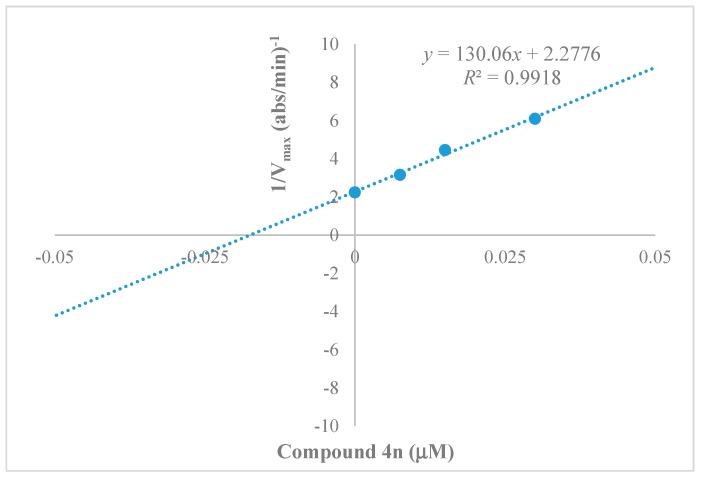
Secondary plot for calculation of the steady-state inhibition constant (K_i_ = 0.0175 µM against BChE) of compound **4n**.

The calculation of the inhibition constant (K_i_) is an effective tool for measuring the affinity of an enzyme for its substrate, and it is easily determined using the secondary plots of the Lineweaver–Burk equation. Hence, a graphical investigation for the lead compound, **4n**, was performed and is presented in Figure 3. It was determined that **4n** has a very close K_i_ value (0.017 µM) to that of tacrine [28], meaning that it is able to firmly bind to the BChE active site.

When the small molecule binds to the BChE catalytic active site (CAS), the type of inhibition of the enzyme is classified as competitive; by contrast, when the small-molecule interacts with the peripheral anionic site (PAS), the enzymatic inhibition is called non-competitive. Furthermore, once the active molecule acts on both CAS and PAS, the enzymatic inhibition is designated as mixed inhibition [29]. In this manner, the enzyme kinetics study suggests that compound **4n** interacts with both BChE functional sites, CAS and PAS [30].

### 2.4. Molecular Docking

So as to explore a possible interacting mode of the lead compound, **4n**, and to evaluate the effects of structural modifications on the BChE enzyme activity, molecular docking was performed and carried out using the X-ray crystal structure of human BChE in complex with tacrine (Protein Data Bank identifier (PDB ID): 4BDS) [31]. Firstly, tacrine was docked in the enzyme’s active site to validate the docking procedure. Thus, the π–π interaction between the aromatic ring of tacrine and the indole of Trp82, and the hydrogen bond between the 9-amino group of tacrine and the carbonyl of His438 were established, as reported in the literature [31].

**Figure 4:**
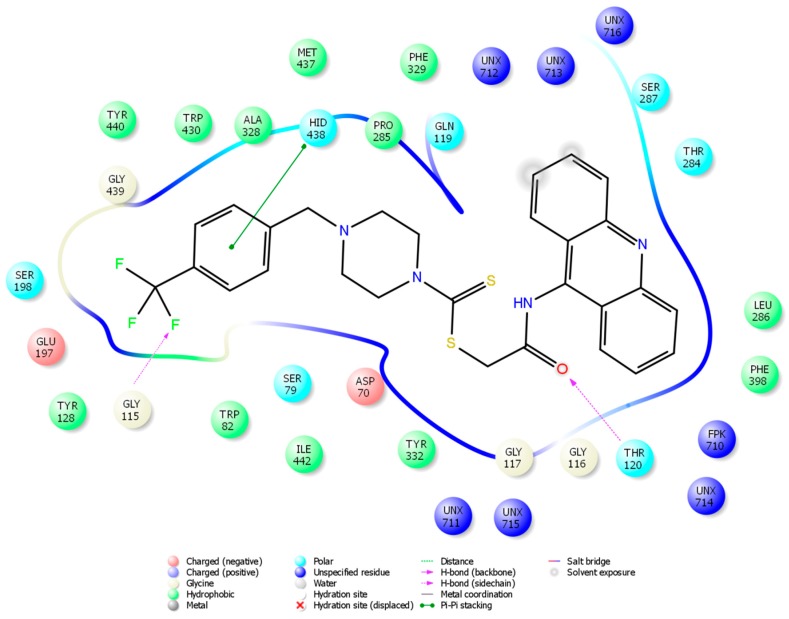
Two-dimensional interaction of compound **4n** with BChE.

**Figure 5:**
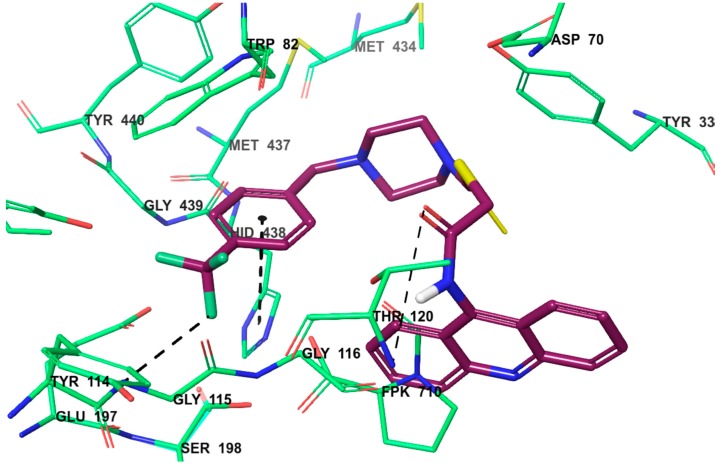
Three-dimensional interaction of compound **4n** with BChE.

The docking poses of compound **4n**, showing two- and three-dimensional interactions, are presented in Figure 4 and Figure 5. According to the poses, there are three main patterns of interaction. The first one is a π–π interaction between the phenyl moiety and the imidazole of His438 in the catalytic site of the BChE enzyme, almost identical to that of tacrine. The second pattern involves a hydrogen bond between the amide carbonyl and the amino group of Thr120. The last and probably the most important interaction is another hydrogen bond between the three-fluoro methyl substituent of **4n** and the amino group of Gly115. These results demonstrate that **4n** has a significant potency to strongly bind to BChE. In particular, the last interaction clarifies why compound **4n** is more active than other derivatives in the series. Findings of the docking analyses confirmed the data reporting that the bulky moieties have good anti-BChE activity due to their binding ability in the active site of BChE [24,25].

### 2.5. MTT Cell Viability Assay and Selectivity Indexes

The active compounds (**4a**, **4b**, **4e**, **4i**, **4m**, **4n**, **4o**, and **4t**) were chosen to determine their cytotoxic activity toward a murine fibroblast healthy cell line (NIH3T3). After incubating the cells with active compounds at concentrations of 0.000316 µM to1000 µM for 24 h, the cell viability was measured using the 3-(4,5-dimethylthiazol-2-yl)-2,5-diphenyltetrazolium (MTT) assay [15]. The inhibition percentage was calculated for each concentration of the test compounds. The IC_50_ values of the compounds were determined using a non-linear regression analysis over the calculated percentage inhibition values, and they are presented in Table 2. Accordingly, the cytotoxicity test proposed that the active derivatives were nontoxic against NIH3T3 cells at their active concentrations against BChE.

### 2.6. BBB Permeability and Drug-Likeness Score (DLS)

BBB permeability is exceptionally fundamental for medications that particularly target and focus on the CNS. The failure of medication particles to penetrate the BBB constitutes a major obstacle for CNS drug candidates and ought to be considered in new drug discovery efforts. For this reason, the BBB permeability of the most active derivatives was computed by a CBLigand-BBB forecast server. This predictor uses two different algorithms, AdaBoost and a support vector machine (SVM), combining with four different fingerprints, employed to predict if a compound can pass (+) or cannot pass (−) the BBB. As presented in Table 3, all calculations for the selected compounds resulted in BBB permeability (+), which is necessary for compounds planning to act as ChE inhibitors [32]. The drug-likeness scores (DLS) were additionally calculated for all active compounds using Molsoft’s chemical fingerprint mode consisting of 5000 marketed drugs from the World Drug Index (positives) and 10,000 carefully selected non-drug compounds (negatives) [33] According to this program, the DLS scores were observed between 1.26 and 2.21, which establishes and fortifies their therapeutic significance in future studies.

## 3. Materials and Methods

### 3.1. Chemistry

All chemicals were purchased from Sigma-Aldrich Chemicals (Sigma-Aldrich Corp., St. Louis, MO, USA) or Merck Chemicals (Merck KGaA, Darmstadt, Germany). Melting points of the synthesized compounds were assessed with an MP90 digital melting point apparatus (Mettler Toledo, Columbus, OH, USA), and were uncorrected. The ^1^H-NMR and ^13^C-NMR spectra were recorded on a Bruker Fourier 300 (Bruker Bioscience, Billerica, MA, USA) in DMSO-*d*_6._ The IR spectra were obtained on a Shimadzu, IR Affinity 1S (Shimadzu, Kyoto, Japan). The mass spectra of the compounds were taken in negative and positive mode using electron spray ionization (ESI) in LCMS/IT/TOF (Shimadzu, Kyoto, Japan) from the solutions of the samples in methanol. The purities of compounds were checked using thin-layer chromatography (TLC) on silica gel 60 F254 (Merck KGaA, Darmstadt, Germany).

#### 3.1.1. General Procedure for the Synthesis of *N*-(9-Acridinyl)-2-Chloroacetamide Derivatives (**2**) 

Chloroacetyl chloride (30 mmol, 2.4 mL) in THF (10 mL) was added dropwise while stirring to a mixture of 9-aminoacridine (**1**; 20 mmol, 3.9 g) and triethylamine (40 mmol, 5.6 mL) in THF (50 mL) at 0–5 °C and stirred for 4 h. The solvent was evaporated under reduced pressure. The residue was washed with water to remove triethylamine hydrochloride, before being dried and crystallized from ethanol [34].

#### 3.1.2. General Procedure for the Synthesis of Sodium *N*-Substituted Piperazine/Morpholine/Piperidine Dithiocarbamates (**3a**–**3u**)

An ethanolic solution (10 mL) of sodium hydroxide (10 mmol, 0.4 g) was added to an ethanolic solution (10 mL) of the secondary amine (10 mmol). The mixture was cooled in an ice bath; additionally, carbon disulfide (100 mmol, 6.0 mL) was added dropwise with continuous stirring for 1 h at room temperature. The precipitates were filtrated and washed with diethyl ether to obtain a white to pale-yellow-colored product in 70–90% yield [14].

#### 3.1.3. General Procedure for the Synthesis of 2-(9-Acridinylamino)-2-Oxoethyl 4-Piperazinyl/Morpholinyl/Piperidinylcarbodithioate Derivatives (**4a**–**4u**) 

An equimolar quantity (5 mmol) of appropriate sodium *N*,*N*-disubstituted dithiocarbamate (**3**) and of *N*-(9-acridinyl)-2-chloroacetamide (**2**) in acetone (10 mL) were refluxed for 8 h. The mixture was cooled, and the precipitate was filtrated. The residue was washed with water, before being dried and crystallized from ethanol to obtain the final products [14].

*2-(9-Acridinylamino)-2-oxoethyl 4-(2-(dimethylamino)ethyl)piperazine-1-carbodithioate* (**4a**). Yield: 88.5%; melting poing (m.p.): 200.6 °C; FTIR (ATR, cm^−1^): 3269 (amide N–H), 2769–2972 (aliphatic C–H), 1662 (C=O), 1463–1506 (C=N and C=C), 1232 (C=S), 756 (out-of-plane C–H bending); ^1^H-NMR (300 MHz, DMSO-*d*_6_; δ, ppm): 2.17–2.18 (6H, d, *J =* 2.4 Hz, N(CH_3_)_2_), 2.43–2.44 (4H, t, *J =* 2.5 Hz, CH_2_–CH_2_), 2.48–2.55 (4H, m, piperazine C_3__,5_–H), 3.97 and 4.25 (4H, two bs, piperazine C_2__,6_–H), 4.62 (2H, s, COCH_2_), 7.57–7.62 (2H, t, *J =* 7.5 Hz, Ar–H), 7.82–7.87 (2H, t, *J =* 7.7 Hz, Ar–H), 8.14–8.17 (2H, d, *J =* 8.7 Hz, Ar–H), 8.25–8.28 (2H, d, *J =* 8.6 Hz, Ar–H), 11.06 (1H, s, –NH–); ^13^C-NMR (75 MHz, DMSO-*d*_6_; δ, ppm): 45.7 (CH_3_), 50.4 (CH_2_), 51.6 (CH_2_), 52.9 (CH_2_), 55.2 (CH_2_), 56.8 (CH_2_), 123.2 (C), 125.4 (CH), 126.2 (CH), 129.6 (CH), 130.9 (CH), 140.6 (C), 149.4 (C-9 in 9-aminoacridine), 167.4 (C=O), 194.9 (C=S); HRMS (*m*/*z*): [M + H]^+^ calculated for C_24_H_29_N_5_OS_2_: 468.1886, found 468.1874.

*2-(9-Acridinylamino)-2-oxoethyl 4-(3-(dimethylamino)propyl)piperazine-1-carbodithioate* (**4b**). Yield: 85.7%; m.p.: 153.8 °C; FTIR (ATR, cm^−1^): 3340 (amide N–H), 2777–2947 (aliphatic C–H), 1653 (C=O), 1411–1458 (C=N and C=C), 1209–1263 (C=S), 758 (out-of-plane C–H bending); ^1^H-NMR (300 MHz, DMSO-*d*_6_; δ, ppm): 1.47–1.56 (2H, m, CH_2_–CH_2_–CH_2_), 2.08 (6H, s, N(CH_3_)_2_), 2.15–2.20 (2H, t, *J =* 7.1 Hz, CH_2_–CH_2_CH_2_N(CH_3_)_2_), 2.24–2.29 (2H, d, *J =* 7.4 Hz, CH_2_CH_2_CH_2_), 2.41–2.42 (4H, d, *J =* 0.9, piperazine C_3__,__5_–H), 3.98 and 4.26 (4H, two s, piperazine C_2__,__6_–H), 4.65 (2H, s, COCH_2_), 7.57–7.62 (2H, q, *J =* 7.6 Hz, Ar–H), 7.81–7.87 (2H, m, Ar–H), 8.14–8.17 (2H, d, *J =* 8.7 Hz, Ar–H), 8.26–8.29 (2H, d, *J =* 8.7 Hz, Ar–H), 11.19 (1H, s, –NH–); ^13^C-NMR (75 MHz, DMSO-*d*_6_; δ, ppm): 24.9 (CH_2_), 45.7 (CH_2_), 50.4 (CH_3_), 51.6 (CH_2_), 52.9 (CH_2_), 55.2 (CH_2_), 56.7 (CH_2_), 123.2 (C), 125.4 (CH), 126.2 (CH), 129.6 (CH), 130.9 (CH), 140.6 (C), 149.4 (C-9 in 9-aminoacridine), 167.4 (C=O), 194.9 (C=S); HRMS (*m*/*z*): [M + H]^+^ calculated for C_25_H_31_N_5_OS_2_: 482.2043, found 482.2047.

*2-(9-Acridinylamino)-2-oxoethyl 4-ethylpiperazine-1-carbodithioate* (**4c**). Yield: 81.0%; m.p.: 221.7 °C; FTIR (ATR, cm^−1^): 3232 (amide N–H), 2358–2974 (aliphatic C–H), 1654 (C=O), 1435–1508 (C=N and C=C), 1219–1271 (C=S), 732–758 (out-of-plane C–H bending); ^1^H-NMR (300 MHz, DMSO-*d*_6_; δ, ppm): 0.98–1.03 (3H, t, *J =* 7.2 Hz, CH_2_CH_3_), 2.33–2.40 (2H, q, *J =* 7.2 Hz, CH_2_CH_3_), 2.47–2.50 (4H, m, piperazine C_3__,5_–H), 3.98 and 4.26 (4H, two s, piperazine C_2__,6_–H), 4.65 (2H, s, COCH_2_), 7.57–7.62 (2H, t, *J =* 7.5 Hz, Ar–H), 7.81–7.87 (2H, t, *J =* 7.6 Hz, Ar–H), 8.14–8.17 (2H, d, *J =* 8.7 Hz, Ar–H), 8.26–8.29 (2H, d, *J =* 8.6 Hz, Ar–H), 11.19 (1H, s, –NH–); ^13^C-NMR (75 MHz, DMSO-*d*_6_; δ, ppm): 12.3 (CH_3_), 50.9 (CH_2_), 51.1 (CH_2_), 51.5 (CH_2_), 52.2 (CH_2_), 123.2 (C), 125.4 (CH), 126.2 (CH), 129.6 (CH), 130.9 (C), 140.7 (C), 149.4 (C-9 in 9-aminoacridine), 167.4 (C=O), 195.0 (C=S); HRMS (*m*/*z*): [M + H] ^+^ calculated for C_22_H_24_N_4_OS_2_: 425.1464, found 425.1459.

*2-(9-Acridinylamino)-2-oxoethyl 4-(4-nitrophenyl)piperazine-1-carbodithioate* (**4d**). Yield: 90.5%; m.p.: 248.2 °C; FTIR (ATR, cm^−1^): 3238 (amide N–H), 2976 (aliphatic C–H), 1670 (C=O), 1417–1598 (C=N and C=C), 1213–1280 (C=S),752 (out-of-plane C–H bending); ^1^H-NMR (300 MHz, DMSO-*d*_6_; δ, ppm): 3.74 (4H, s, piperazine C_3__,5_–H), 4.19 and 4.42 (4H, two bs, piperazine C_2__,6_–H), 4.68 (2H, s, COCH_2_), 6.93–6.96 (2H, d, *J =* 9.5 Hz, Ar–H), 7.59–7.63 (2H, t, *J =* 6.6 Hz, Ar–H), 7.82–7.87 (2H, t, *J =* 7.7 Hz, Ar–H), 8.07–8.10 (2H, d, *J =* 9.4 Hz, Ar–H), 8.14–8.17 (2H, d, *J =* 8.7 Hz, Ar–H), 8.27–8.30 (2H, d, *J =* 8.5 Hz, Ar–H), 11.10 (1H, s, –NH–); ^13^C-NMR (75 MHz, DMSO-*d*_6_; δ, ppm): 45.2 (CH_2_), 48.9 (CH_2_), 50.6 (CH_2_), 112.3 (C), 123.2 (CH), 125.4 (CH), 126.2 (CH), 126.3 (CH), 129.6 (CH), 130.9 (CH), 137.3 (C), 140.6 (C), 149.4 (C), 154.2 (C-9 in 9-aminoacridine), 167.3 (C=O), 195.4 (C=S); HRMS (*m*/*z*): [M + H]^+^ calculated for C_26_H_23_N_5_O_3_S_2_: 518.1315, found 518.1299.

*2-(9-Acridinylamino)-2-oxoethyl 4-(2-hydroxyethyl)piperazine-1-carbodithioate* (**4e**). Yield: 84.4%; m.p.: 219.2 °C; FTIR (ATR, cm^−1^): 3226 (amide N–H), 2978 (aliphatic C–H), 1660 (C=O), 1433–1558 (C=N and C=C), 1228 (C=S), 758 (out-of-plane C–H bending); ^1^H-NMR (300 MHz, DMSO-*d*_6_; δ, ppm): 2.49–2.50 (2H, t, *J =* 1.7 Hz, OH–CH_2_–CH_2_–), 2.61 (4H, s, piperazine C_3__,5_–H), 3.54 (2H, s, OH–CH_2_–CH_2_–), 4.00 and 4.28 (4H, s, piperazine C_2__,6_–H), 4.64 (2H, s, COCH_2_), 7.58–7.62 (2H, t, *J =* 7.4 Hz, Ar–H), 7.82–7.87 (2H, t, *J =* 7.4 Hz, Ar–H), 8.14–8.17 (2H, d, *J =* 8.6 Hz, Ar–H), 8.26–8.28 (2H, d, *J =* 8.6 Hz, Ar–H), 11.10 (1H, s, –NH–); ^13^C-NMR (75 MHz, DMSO-*d*_6_; δ, ppm): 50.2 (CH_2_), 51.4 (CH_2_), 52.9 (CH_2_), 58.8 (CH_2_), 59.8 (CH_2_), 123.2 (C), 125.4 (CH), 126.2 (CH), 129.6 (CH), 130.9 (CH), 140.6 (C), 149.4 (C-9 in 9-aminoacridine), 167.4 (C=O), 195.1 (C=S); HRMS (*m*/*z*): [M + H] ^+^ calculated for C_22_H_24_N_4_O_2_S_2_: 441.1413, found 441.1406.

*2-(9-Acridinylamino)-2-oxoethyl 4-phenylpiperazine-1-carbodithioate* (**4f**). Yield: 94.5%; m.p.: oil; FTIR (ATR, cm^−1^): 3352 (amide N–H), 2881 (aliphatic C–H), 1660 (C=O), 1417–1506 (C=N and C=C), 1228 (C=S), 756–883 (out-of-plane C–H bending); ^1^H-NMR (300 MHz, DMSO-*d*_6_; δ, ppm): 3.31–3.35 (4H, d, *J* =9.4 Hz, piperazine C_3__,5_–H), 4.15 and 4.42 (4H, two bs, piperazine C_2__,6_–H), 4.66 (2H, s, COCH_2_), 6.78–6.83 (1H, t, *J =* 7.2 Hz, Ar–H), 6.94–6.97 (2H, d, *J =* 8.0 Hz, Ar–H), 7.21–7.26 (2H, t, *J =* 7.9 Hz, Ar–H), 7.58–7.63 (2H, t, *J =* 7.6 Hz, Ar–H), 7.82–7.86 (2H, t, *J* = 6.7 Hz, Ar–H), 8.14–8.17 (2H, d, *J =* 8.7 Hz, Ar–H), 8.27–8.30 (2H, d, *J =* 8.5 Hz, Ar–H), 11.08 (1H, s, –NH–); ^13^C-NMR (75 MHz, DMSO-*d*_6_; δ, ppm): 48.1 (CH_2_), 50.1 (CH_2_), 51.3 (CH_2_), 115.9 (C), 119.7 (CH), 123.2 (CH), 125.4 (CH), 126.2 (CH), 129.5 (CH), 129.6 (CH), 130.9 (CH), 140.6 (C), 149.4 (C), 150.5 (C-9 in 9-aminoacridine), 167.4 (C=O), 195.3 (C=S); HRMS (*m*/*z*): [M + H] ^+^ calculated for C_26_H_24_N_4_OS_2_: 473.1464, found 473.1471.

*2-(9-Acridinylamino)-2-oxoethyl 4-methylpiperazine-1-carbodithioate* (**4g**). Yield: 95.3%; m.p.: 243.2 °C; FTIR (ATR, cm^−1^): 3226 (amide N–H), 2783–2976 (aliphatic C–H), 1651 (C=O), 1435–1571(C=N and C=C), 1224–1288 (C=S), 758 (out-of-plane C–H bending); ^1^H-NMR (300 MHz, DMSO-*d*_6_; δ, ppm): 2.21 (3H, s, CH_3_), 2.42–2.45 (4H, t, *J =* 4.5 Hz, piperazine C_3__,5_–H), 3.99 and 4.27 (4H, two s, piperazine C_2__,6_–H), 4.61 (2H, s, COCH_2_), 7.58–7.63 (2H, t, *J =* 7.5 Hz, Ar–H), 7.82–7.87 (2H, t, *J =* 7.3 Hz, Ar–H), 8.15–8.18 (2H, d, *J =* 8.7 Hz, Ar–H), 8.25–8.28 (2H, d, *J =* 8.6 Hz, Ar–H), 10.94 (1H, s, –NH–); ^13^C-NMR (75 MHz, DMSO-*d*_6_; δ, ppm): 45.6 (CH_2_), 50.3 (CH_3_), 51.6 (CH_2_), 54.5 (CH_2_), 123.2 (CH), 125.3 (CH), 126.3 (CH), 129.3 (CH), 130.9 (CH), 140.5 (CH), 149.4 (C-9 in 9-aminoacridine), 167.4 (C=O), 195.1 (C=S); HRMS (*m*/*z*): [M + H]^+^ calculated for C_21_H_22_N_4_OS_2_: 411.1308, found 411.1307.

*2-(9-Acridinylamino)-2-oxoethyl 4-(4-methoxyphenyl)piperazine-1-carbodithioate* (**4h**). Yield: 89.4%; m.p.: 243.8 °C; FTIR (ATR, cm^−1^): 3284 (amide N–H), 3057–2945 (aliphatic C–H), 1664 (C=O), 1431–1508 (C=N and C=C), 1215 (C=S), 707–756 (out-of-plane C–H bending), ^1^H-NMR (300 MHz, DMSO-*d*_6_; δ, ppm): 3.16–3.35 (4H, s, piperazine C_3,5_–H), 3.68 (3H, s, OCH_3_), 4.14 and 4.42 (4H, two s, piperazine C_2,6_–H), 4.66 (2H, s, COCH_2_), 6.81–6.84 (2H, d, *J =* 8.5 Hz, Ar–H), 6.90–6.93 (2H, d, *J =* 8.4 Hz, Ar–H), 7.59–7.64 (2H, t, *J =* 7.3 Hz, Ar–H), 7.82–7.87 (2H, t, *J =* 7.4 Hz, Ar–H), 8.16–8.18 (2H, d, *J =* 8.6 Hz, Ar–H), 8.27–8.30 (2H, d, *J =* 8.6 Hz, Ar–H), 10.97 (1H, s, –NH–); ^13^C-NMR (75 MHz, DMSO-*d*_6_; δ, ppm): 49.8 (CH_2_), 51.4 (CH_2_), 55. 7 (CH_3_), 60.21 (CH_2_), 114.8 (C), 118.7 (CH), 123.2 (CH), 125.3 (CH), 126.3 (CH), 129.6 (CH), 130.9 (CH), 140.5 (C), 144.9 (C), 149.4 (C), 153.9 (C-9 in 9-aminoacridine), 167.4 (C=O), 195.3 (C=S); HRMS (*m*/*z*): [M + H] ^+^ calculated for C_27_H_26_N_4_O_2_S_2_: 503.1570, found 503.1547.

*2-(9-Acridinylamino)-2-oxoethyl 4-(4-chlorophenyl)piperazine-1-carbodithioate* (**4i**). Yield: 97.0%; m.p.: 213.6 °C; FTIR (ATR, cm^−1^): 3223 (amide N–H), 2706–2912 (aliphatic C–H), 1672 (C=O), 1417–1573 (C=N and C=C), 1226 (C=S), 815 (para aromatic substitution), 756 (out-of-plane C–H bending); ^1^H-NMR (300 MHz, DMSO-*d*_6_; δ, ppm): 2.44–2.50 (4H, q, *J =* 5.8 Hz, piperazine C_3,5_–H), 4.02 and 4.45 (4H, two bs, piperazine C_2,6_–H), 4.60 (2H, s, COCH_2_), 6.97–7.00 (2H, d, *J =* 9.1 Hz, Ar–H), 7.25–7.28 (2H, d, *J =* 9.0 Hz, Ar–H), 7.57–7.62 (2H, t, *J =* 7.5 Hz, Ar–H), 7.81–7.85 (2H, t, *J =* 5.9 Hz, Ar–H), 8.14–8.17 (2H, d, *J =* 8.7 Hz, Ar–H), 8.27–8.30 (2H, d, *J =* 8.6 Hz, Ar–H), 11.22 (1H, s, –NH–); ^13^C-NMR (75 MHz, DMSO-*d*_6_; δ, ppm): 45.6 (CH_2_), 49.8 (CH_2_), 51.0 (CH_2_), 117.3 (CH), 118.0 (CH), 123.2 (CH), 125.2 (CH), 126.2 (CH), 129.2 (CH), 129.3 (CH), 129.6 (CH), 130.9 (CH), 140.7 (C), 149.4 (C-9 in 9-aminoacridine), 167.4 (C=O), 195.3 (C=S); HRMS (*m*/*z*): [M + H] ^+^ calculated for C_26_H_23_ClN_4_OS_2_: 507.1075, found 507.1069.

*2-(9-Acridinylamino)-2-oxoethyl 4-(pyrimidin-2-yl)piperazine-1-carbodithioate* (**4j**). Yield: 84.3%; m.p.: 248.4 °C; FTIR (ATR, cm^−1^): 3263 (amide N–H), 2852–2897 (aliphatic C–H), 1662 (C=O), 1425–1583 (C=N and C=C), 1222–1257 (C=S), 759–792 (out-of-plane C–H bending); ^1^H-NMR (300 MHz, DMSO-*d*_6_; δ, ppm): 3.91 (4H, s, piperazine C_3,5_–H), 4.13 and 4.38 (4H, two s, piperazine C_2,6_–H), 4.68 (2H, s, COCH_2_), 6.67–6.70 (1H, t, *J =* 4.7 Hz, Ar–H), 7.58–7.63 (2H, t, *J =* 7.6 Hz, Ar–H), 7.82–7.87 (2H, t, *J =* 7.4 Hz, Ar–H), 8.14–8.18 (2H, d, *J =* 8.7 Hz, Ar–H), 8.27–8.30 (2H, d, *J =* 8.6 Hz, Ar–H), 8.39–8.41 (2H, d, *J =* 4.7 Hz, Ar–H), 11.16 (1H, s, –NH–); ^13^C-NMR (75 MHz, DMSO-*d*_6_; δ, ppm): 43.0 (CH_2_), 49.8 (CH_2_), 51.2 (CH_2_), 111.1 (C), 123.2 (CH), 125.4 (CH), 126.2 (CH), 129.6 (CH), 130.9 (CH), 140.6 (C), 149.4 (CH), 158.48 (C), 161.3 (C-9 in 9-aminoacridine), 167.4 (C=O), 195.4 (C=S). HRMS (*m*/*z*): [M + H] ^+^ calculated for C_24_H_22_N_6_OS_2_: 475.1369, found 475.1366.

*2-(9-Acridinylamino)-2-oxoethyl 4-benzylpiperazine-1-carbodithioate* (**4k**). Yield: 91.4%; m.p.: 222.3 °C; FTIR (ATR, cm^−1^): 3248 (amide N–H), 2818–2908 (aliphatic C–H), 1668 (C=O), 1417–1510 (C=N and C=C), 1230 (C=S), 731–756 (out-of-plane C–H bending); ^1^H-NMR (300 MHz, DMSO-*d*_6_; δ, ppm): 3.53 and 3.99 (4H, two bs, piperazine C_2_,_6_–H), 4.28 (2H, s, C_6_H_5_–CH_2_–), 4.62 (2H, s, COCH_2_), 7.26–7.32 (5H, m, Ar–H), 7.57–7.62 (2H, t, *J =* 7.5 Hz, Ar–H), 7.82–7.87 (2H, t, *J =* 7.5 Hz, Ar–H), 8.14–8.17 (2H, d, *J =* 8.7 Hz, Ar–H), 8.25–8.28 (2H, d, *J =* 8.6 Hz, Ar–H), 11.05 (1H, s, –NH–); ^13^C-NMR (75 MHz, DMSO-*d*_6_; δ, ppm): 50.4 (CH_2_), 51.7 (CH_2_), 52.4 (CH_2_), 61.8 (CH_2_), 123.2 (C), 125.3 (CH), 126.2 (CH), 127.6 (CH), 128.7 (CH), 129.4 (CH), 129.6 (CH), 130.9 (CH), 138.0 (C), 140.6 (C), 149.4 (C-9 in 9-aminoacridine), 167.4 (C=O), 195.0 (C=S); HRMS (*m*/*z*): [M + H]^+^ calculated for C_27_H_26_N_4_OS_2_: 487.1621, found 487.1622.

*2-(9-Acridin-ylamino)-2-oxoethyl 4-cyclohexylpiperazine-1-carbodithioate* (**4l**). Yield: 88.4%; m.p.: 225.4°C; FTIR (ATR, cm^−1^): 3309 (amide N–H), 2357–2931 (aliphatic C–H), 1676 (C=O), 1469–1566 (C=N and C=C), 1230–1274 (C=S), 754–866 (out-of-plane C–H bending); ^1^H-NMR (300 MHz, DMSO-*d*_6_; δ, ppm): 1.03–1.18 (6H, m, cyclohexyl –CH_2_–), 1.54–1.57 (1H, d, *J =* 11.2 Hz, cyclohexyl –CH–), 1.72 (4H, s, cyclohexyl –CH_2_–), 2.60 (4H, s, piperazine C_3,5_–H), 3.94 and 4.24 (4H, two bs, piperazine C_2,6_–H), 4.62 (2H, s, COCH_2_), 7.57–7.62 (2H, t, *J =* 7.5 Hz, Ar–H), 7.82–7.87 (2H, t, *J =* 7.6 Hz, Ar–H), 8.14–8.17 (2H, d, *J =* 8.7 Hz, Ar–H), 8.25–8.28 (2H, d, *J =* 8.6 Hz, Ar–H), 11.04 (1H, s, –NH–); ^13^C-NMR (75 MHz, DMSO-*d*_6_; δ, ppm): 25.7 (CH_2_), 26.3 (CH_2_), 28.8 (CH_2_), 48.6 (CH_2_), 50.9 (CH_2_), 52.2 (CH_2_), 62.7 (CH), 123.2 (C), 125.4 (CH), 126.2 (CH), 129.6 (CH), 130.9 (CH), 140.6 (C), 149.4 (C-9 in 9-aminoacridine), 167.4 (C=O), 194.7 (C=S); HRMS (*m*/*z*): [M + H]^+^ calculated for C_26_H_30_N_4_OS_2_: 479.1934, found 479.1929.

*2-(9-Acridinylamino)-2-oxoethyl 4-benzhydrylpiperazine-1-carbodithioate* (**4m**). Yield: 84.3%; m.p.: 137.0 °C; FTIR (ATR, cm^−1^): 3246 (amide N–H), 2355–2920 (aliphatic C–H), 1656 (C=O), 1427–1571 (C=N and C=C), 1226–1284 (C=S), 705–758 (out-of-plane C–H bending); ^1^H-NMR (300 MHz, DMSO-*d*_6_; δ, ppm): 2.44–2.50 (4H, d, *J =* 5.8 Hz, piperazine C_3,5_–H), 4.02 and 4.28 (4H, two s, piperazine C_2,6_–H), 4.40 (1H, s, C_12_H_10_–CH–), 4.60 (2H, s, COCH_2_), 7.18–7.23 (3H, t, *J =* 7.0 Hz, Ar–H), 7.29–7.32 (4H, t, *J =* 7.4 Hz, Ar–H), 7.44 (4H, s, Ar–H), 7.57–7.62 (2H, t, *J =* 7.6 Hz, Ar–H), 7.82–7.88 (2H, m, Ar–H), 7.15–7.18 (2H, d, *J =* 8.7 Hz, Ar–H), 7.24–7.27 (2H, d, *J =* 8.6 Hz, Ar–H), 11.02 (1H, s, –NH–); ^13^C-NMR (75 MHz, DMSO-*d*_6_; δ, ppm): 51.4 (CH_2_), 60.7 (CH_2_), 72.7 (CH_2_), 74.6 (CH), 117.9 (C), 123.1 (CH), 125.4 (CH), 126.3 (CH), 127.6 (CH), 128.1 (CH), 129.1 (CH), 131.2 (CH), 140.9 (C), 142.6 (C), 149.1 (C-9 in 9-aminoacridine), 167.4 (C=O), 195.1 (C=S); HRMS (*m*/*z*): [M + H]^+^ calculated for C_33_H_30_N_4_OS_2_: 563.1934, found 563.1924.

*2-(9-Acridinylamino)-2-oxoethyl 4-(4-(trifluoromethyl)benzyl)piperazine-1-carbodithioate* (**4n**). Yield: 87.7%; m.p.: 161.5 °C; FTIR (ATR, cm^−1^): 3232 (amide N–H), 2814–2920 (aliphatic C–H), 1653 (C=O), 1415–1510 (C=N and C=C), 1219 (C=S), 756 (out-of-plane C–H bending); ^1^H-NMR (300 MHz, DMSO-*d*_6_; δ, ppm): 2.48–2.52 (4H, t, *J =* 5.10 Hz, piperazine C_3,5_–H), 3.62 and 4.00 (4H, two s, piperazine C_2,6_–H), 4.29 (2H, s, C_6_H_5_–CH_2_–), 4.62 (2H, s, COCH_2_), 7.55–7.57 (2H, d, *J =* 7.9 Hz, Ar–H), 7.60–7.62 (2H, d, *J* = 7.7 Hz, Ar–H), 7.68–7.71 (2H, d, *J =* 8.2 Hz Ar–H), 7.82–7.87 (2H, t, *J =* 7.8 Hz, Ar–H), 7.14–7.17 (2H, d, *J =* 8.8 Hz, Ar–H), 7.25–7.27 (2H, d, *J =* 8.6 Hz, Ar–H), 11.01 (1H, s, –NH–); ^13^C-NMR (75 MHz, DMSO-*d*_6_; δ, ppm): 52.4 (CH_2_), 60.8 (CH_2_), 72.7 (CH_2_), 125.0 (q, *J =* 214.9 Hz, CF_3_), 123.2 (CH), 125.3 (CH), 125.6 (q, *J* =3.6 Hz, trifluoromethylphenyl C_3,3′_), 126.2 (CH), 129.6 (CH), 129.9 (q, *J* = 39.8 Hz, trifluoromethylphenyl C_4_), 130.0 (CH), 130.1 (C), 140.6 (C), 143.2 (C), 149.4 (C-9 in 9-aminoacridine), 167.4 (C=O), 195.1 (C=S); HRMS (*m*/*z*): [M + H]^+^ calculated for C_28_H_25_F_3_ N_4_OS_2_: 555.1495, found 555.1493.

*2-(9-Acridinylamino)-2-oxoethyl 4-(4-methylbenzyl)piperazine-1-carbodithioate* (**4o**). Yield: 91.0%; m.p.: 205.8 °C; FTIR (ATR, cm^−1^): 3255 (amide N–H), 2792–2902 (aliphatic C–H), 1654 (C=O), 1435–1508 (C=N and C=C), 1220 (C=S), 759 (out-of-plane C–H bending); ^1^H-NMR (300 MHz, DMSO-*d*_6_; δ, ppm): 2.27 (3H, s, –CH_3_), 2.47–2.50 (4H, m, piperazine C_3,5_–H), 3.47 (2H, s,C_6_H_5_–CH_2_), 3.98 and 4.26 (4H, two s, piperazine C_2,6_–H), 4.63 (2H, s, COCH_2_), 7.11–7.14 (2H, d, *J =* 7.9 Hz, Ar–H), 7.18–7.20 (2H, d, *J* =7.9 Hz, Ar–H), 7.56–7.61 (2H, t, *J =* 7.5 Hz, Ar–H), 7.86–7.81 (2H, t, *J =* 7.4 Hz, Ar–H), 8.14–8.17 (2H, d, *J =* 8.7 Hz, Ar–H), 8.25–8.28 (2H, d, *J =* 8.6 Hz, Ar–H), 11.15 (1H, s, –NH–); ^13^C-NMR (75 MHz, DMSO-*d*_6_; δ, ppm): 21.2 (CH_3_), 50.4 (CH_2_), 51.6 (CH_2_), 52.4 (CH_2_), 61.5 (CH_2_), 123.2 (C), 125.4 (CH), 126.2 (CH), 129.3 (CH), 129.4 (CH), 129.6 (CH), 130.9 (CH), 134.8 (C), 36.7 (C), 140.6 (C), 149.4 (C-9 in 9-aminoacridine), 167.4 (C=O), 195.0 (C=S); HRMS (*m*/*z*): [M + H]^+^ calculated for C_28_H_28_N_4_OS_2_: 501.1777, found 501.1774.

*2-(9-Acridinylamino)-2-oxoethyl 4-(4-fluorophenyl)piperazine-1-carbodithioate* (**4p**). Yield: 96.4%; m.p.: 227.7 °C; FTIR (ATR, cm^−1^): 3257 (amide N–H), 2800–2906 (aliphatic C–H), 1653 (C=O), 1433 (C=N and C=C), 1213 (C=S), 1029 (C–F), 825 (1,4-disubstituted benzene), 756 (out-of-plane C–H bending); ^1^H-NMR (300 MHz, DMSO-*d*_6_; δ, ppm): 3.24–3.27 (4H, t, *J =* 4.8 Hz, piperazine C_3__,5_–H), 4.16 and 4.39 (4H, two s, piperazine C_2__,6_–H), 4.67 (2H, s,COCH_2_), 6.94–6.99 (2H, m, Ar–H), 7.04–7.10 (2H, t, *J =* 8.9 Hz, Ar–H), 7.58–7.63 (2H, t, *J =* 7.6 Hz, Ar–H), 7.82–7.87 (2H, t, *J =* 7.7 Hz, Ar–H), 8.15–8.18 (2H, d, *J =* 8.7 Hz, Ar–H), 8.27–8.30 (2H, d, *J =* 8.6 Hz, Ar–H), 11.07 (1H, s, –NH–); ^13^C-NMR (75 MHz, DMSO-*d*_6_; δ, ppm): 49.0 (CH_2_), 50.1 (CH_2_), 51.36 (CH_2_), 115.9 (d, *J* =21.8 Hz, fluorophenyl C_3,3′_), 117.9 (d, *J* = 7.5 Hz, fluorophenyl C_2,2′_), 123.2 (CH), 125.4 (CH), 126.2 (CH), 129.6 (CH), 130.9 (CH), 140.6 (CH), 147.4 (d, *J* = 2.3 Hz, fluorophenyl C_1_), 149.4 (C-9 in 9-aminoacridine), 156.8 (d, *J* = 234.8 Hz, fluorophenyl C_4_), 167.4 (C=O), 195.3 (C=S); HRMS (*m*/*z*): [M + H]^+^ calculated for C_26_H_23_FN_4_OS_2_: 490.62, found 491.13; HRMS (*m*/*z*): [M + H]^+^ calculated for C_26_H_23_FN_4_OS_2_: 491.1370, found 491.1354.

*2-(9-Acridinylamino)-2-oxoethyl 4-(furan-2-carbonyl)piperazine-1-carbodithioate* (**4q**). Yield: 95.5%; m.p.: 218.9 °C; FTIR (ATR, cm^−1^): 3255 (amide N–H), 2895–2995 (aliphatic C–H), 1653 (furan-2-carbonyl), 1616 (amide C=O), 1423 (C=N and C=C), 1215 (C=S), 758 (out-of-plane C–H bending); ^1^H-NMR (300 MHz, DMSO-*d*_6_; δ, ppm): 3.88 (4H, s, piperazine C_3,5_–H), 4.12 and 4.36 (4H, two s, piperazine –CH_2_), 4.68 (2H, s, COCH_2_), 6.64–6.65 (1H, m, Ar–H), 7.07–7.08 (2H, d, *J* = 3.4 Hz, Ar–H), 7.57–7.63 (2H, t, *J =* 7.6 Hz, Ar–H), 7.81–7.87 (3H, t, *J =* 8.1 Hz, Ar–H), 8.14–8.17 (2H, d, *J =* 8.7 Hz, Ar–H), 8.27–8.30 (2H, d, *J =* 8.6 Hz, Ar–H), 11.21 (1H, s, –NH–); ^13^C-NMR (75 MHz, DMSO-*d*_6_; δ, ppm): 49.6 (CH_2_), 51.1 (CH_2_), 111.9 (C), 116.6 (CH), 123.2 (CH), 125.4 (CH), 126.2 (CH), 129.6 (CH), 130.9 (CH), 140.6 (CH), 145.6 (C), 147.1 (C), 149.4 (C), 159.0 (C-9 in 9-aminoacridine), 167.4 (C=O), 195.7 (C=S); HRMS (*m*/*z*): [M + H]^+^ calculated for C_25_H_22_N_4_O_3_S_2_: 491.1206, found 491.1186.

*2-(9-Acridinylamino)-2-oxoethyl morpholine-4-carbodithioate* (**4r**). Yield: 89.2%; m.p.: 237.8 °C; FTIR (ATR, cm^−1^): 3232 (amide N–H), 2864–2949 (aliphatic C–H), 1653 (amide C=O), 1417–1423 (C=N and C=C), 1273 (C=S), 1001 (C–O–C of morpholine), 864–758 (out-of-plane C–H bending); ^1^H-NMR (300 MHz, DMSO-*d*_6_; δ, ppm): 3.71–3.72 (4H, d, *J* = 4.3 Hz, morpholine C_3.5_–H), 4.01 and 4.25 (4H, two bs, morpholine C_2,6_–H), 4.65 (2H, s, COCH_2_), 7.58–7.63 (2H, t, *J* = 7.5 Hz, Ar–H), 7.82–7.87 (2H, t, *J =* 7.6 Hz, Ar–H), 8.15–8.18 (2H, d, *J =* 8.7 Hz, Ar–H), 8.26–8.29 (2H, d, *J =* 8.6 Hz, Ar–H), 11.06 (1H, s, –NH–); ^13^C-NMR (75 MHz, DMSO-*d*_6_; δ, ppm): 50.9 (CH_2_), 51.8 (CH_2_), 66.1 (CH_2_), 123.2 (C), 125.3 (CH), 126.2 (CH), 129.6 (CH), 130.9 (CH), 140.6 (C), 149.4 (C-9 in 9-aminoacridine), 167.4 (C=O), 195.7 (C=S); HRMS (*m*/*z*): [M + H]^+^ calculated for C_20_H_19_N_3_O_2_S_2_: 398.0991, found 398.0977.

*2-(9-Acridinylamino)-2-oxoethyl piperidine-1-carbodithioate* (**4s**). Yield: 84.4%; m.p.: 265.4 °C; FTIR (ATR, cm^−1^): 3255 (amide N–H), 2904–2983 (aliphatic C–H), 1653 (amide C=O), 1417 (C=N and C=C), 1217 (C=S), 1001 (C–N of piperidine), 758 (out-of-plane C–H bending); ^1^H-NMR (300 MHz, DMSO-*d*_6_; δ, ppm): 1.64 (6H, s, piperidine –CH_2_), 3.96 and 4.25 (4H, two s, piperidine CH_2_–N–CH_2_), 4.62 (2H, s, COCH_2_), 7.57–7.61 (2H, t, *J =* 6.6 Hz, Ar–H), 7.82–7.86 (2H, t, *J =* 6.9 Hz, Ar–H), 8.14–8.16 (2H, d, *J =* 8.3 Hz, Ar–H), 8.26–8.29 (2H, d, *J =* 8.5 Hz, Ar–H), 11.14 (1H, s, –NH–); ^13^C-NMR (75 MHz, DMSO-*d*_6_; δ, ppm): 23.9 (CH_2_), 26.0 (CH_2_), 51.7 (CH_2_), 53.1 (CH_2_), 123.2 (C), 125.4 (CH), 126.2 (CH), 129.6 (CH), 130.9(CH), 140.7 (C), 149.4 (C-9 in 9-aminoacridine), 167.5 (C=O), 193.7 (C=S); HRMS (*m*/*z*): [M + H]^+^ calculated for C_21_H_21_N_3_OS_2_: 396.1199, found 396.1184.

*2-(9-Acridinylamino)-2-oxoethyl 2-methylpiperidine-1-carbodithioate* (**4t**). Yield: 93.3%; m.p.: 265.4 °C; FTIR (ATR, cm^−1^): 3255 (amide N–H), 2924 (aliphatic C–H), 1653 (amide C=O), 1417–1463 (C=N and C=C), 1215–1263 (C=S), 1001 (C–N of piperidine), 758 (out-of-plane C–H bending); ^1^H-NMR (300 MHz, DMSO-*d*_6_; δ, ppm): 1.19–1.25 (9H, m, piperidine –CH_2_, –CH_3_), 4.26–4.30 (3H, m, piperidine –CH_2_, –CH–), 4.61 (2H, s, COCH_2_), 7.60 (2H, t, *J =* 7.5 Hz, Ar–H), 7.85 (2H, t, *J =* 7.5 Hz, Ar–H), 8.16 (2H, d, *J =* 8.6 Hz, Ar–H), 8.28 (2H, d, *J =* 8.7 Hz, Ar–H), 10.94 (1H, s, –NH–); ^13^C-NMR (75 MHz, DMSO-*d*_6_; δ, ppm): 18.5 (CH_3_), 18.7 (CH_2_), 25.7 (CH_2_), 26.2 (CH_2_), 52.2 (CH_2_), 54.6 (CH), 123.2 (C), 125.4 (CH), 126.2 (CH), 129.6 (CH), 130.9 (CH), 140.3 (C), 149.4 (C-9 in 9-aminoacridine), 167.6 (C=O), 194.3 (C=S); HRMS (*m*/*z*): [M + H]^+^ calculated for C_22_H_23_N_3_OS_2_: 410.1355, found 410.1338.

*2-(9-Acridinylamino)-2-Oxoethyl 4-Benzylpiperidine-1-Carbodithioate* (**4u**). Yield: 90.7%; m.p.: 265.4 °C; FTIR (ATR, cm^−1^): 3261 (amide N–H), 2852–2922 (aliphatic C–H), 1653 (amide C=O), 1452–1570 (C=N and C=C), 1261 (C=S), 1016 (C–N of piperidine), 758 (out-of-plane C–H bending); ^1^H-NMR (300 MHz, DMSO-*d*_6_; δ, ppm): 1.22–1.26 (5H, m, piperidine –CH_2_, –CH), 3.04–3.08 (2H, m, piperidine –CH_2_–), 3.20–3.25 (2H, m, piperidine –CH_2_–), 3.97 (2H, s, C_6_H_5_–CH_2_–), 4.61 (2H, s, COCH_2_), 7.58 (2H, t, *J =* 7.6 Hz, Ar–H), 7.67 (2H, t, *J =* 7.5 Hz, Ar–H), 7.77–7.86 (5H, m, benzyl –CH), 8.15 (2H, d, *J =* 8.7 Hz, Ar–H), 8.28 (2H, d, *J =* 8.7 Hz, Ar–H); ^13^C-NMR (75 MHz, DMSO-*d*_6_; δ, ppm): 37.1 (CH), 42.0 (CH_2_), 48.7 (CH_2_), 50.6 (CH_2_), 54.6 (CH_2_), 122.4 (C), 125.5 (CH), 126.0 (CH), 126.3 (CH), 128.7 (CH), 129.5 (CH), 130.3 (CH), 130.9 (CH), 132.2 (CH), 140.3 (C), 149.2 (C-9 in 9-aminoacridine), 167.6 (C=O), 193.9 (C=S); HRMS (*m*/*z*): [M + H]^+^ calculated for C_28_H_27_N_3_OS_2_: 486.1668, found 486.1654.

### 3.2. AChE/BChE Activity Assay

All enzymes were obtained from Sigma-Aldrich (Steinheim, Germany). Ellman’s reagent and the substrates were obtained from Fluka (Buchs, Switzerland). The chemicals used to prepare the buffer solution were purchased from Merck (Darmstadt, Germany).

The enzyme inhibitory activities of the synthesized compounds were investigated by applying Ellman’s method [35]. Enzyme solutions were dissolved in gelatin solution (1%; 2.5 units/mL). Compounds **4a**–**4u** and reference agents were prepared in 2% DMSO at concentrations of 10^−3^ M and 10^−4^ M. The enzyme solution (20 µL/well) and inhibitor solution (20 µL/well) were mixed with buffer (140 µL/well, pH 8 ± 0.1) and incubated at 25 °C for 5 min. Then, the reaction was initiated by the addition of Ellman’s reagent 5,5′-Dithiobis(2-nitrobenzoic acid) (DTNB; 20 µL/well, 10 mM) and the substrate (10 µL/well, 75 mM). The absorbance was measured for 10 min at 412 nm. The enzyme solution was processed as a control. All readings were adjusted with blank-reading. All assays were performed in four independent wells. The same procedure was applied for further concentrations (10^−5^–10^−9^ M) of reference agents and synthesized compounds displaying ≥50% inhibition at initial concentrations (10^−3^ and 10^−4^ M). The IC_50_ values were calculated by applying regression analyses using Microsoft Excel 2013 [36]. Absorbance differences between the two readings were taken, and percentage inhibition rates were calculated according to the following formula:

$$\;\%\;Inhibition=\frac{\left[\left(A\left(C\right)-A\left(B\right)\right)-\left(A\left(I\right)-A\left(B\right)\right)\right]}{\left(A\left(C\right)-A\left(B\right)\right)}\times 100.\;$$

**Blank (*B*)**: the well in which the inhibitor compound and substrate were not added;
**Control (*C*)**: the well where the inhibitor compound was not added;
***A*(*B*)**: the difference in absorbance reading for the blank;
***A*(*C*)**: the difference in absorbance reading for the control;
***A*(*I*)**: the difference in absorbance reading for the inhibitor compounds.

### 3.3. Enzyme Kinetics

In the kinetics studies, the assay protocol specified for the inhibition assay was identical. However, unlike the inhibition method, the concentrations of the most active inhibitor compound, **4n**, were used at the calculated IC_50_/2, IC_50_, and 2 × IC_50_ values. The substrate BTCI solution with 10 serial dilutions at different concentrations in the range of 150–0.2929 mM were prepared and used. Measurements were performed one of two ways, in the presence and in the absence of the inhibitor. Firstly, the solutions of compound **4n** at three different concentrations were added to the wells (20 μL/well). After addition of BChE (20 μL/well), the mixture was incubated under the same conditions as the activity assay. Then, the reaction was initiated by the addition of Ellman’s reagent (DTNB; 20 µL/well, 10 mM) and the substrate (10 µL/well, 75 mM). The absorbance was measured for 10 min at 412 nm. The enzyme solution was processed as a control. All readings were adjusted with blank-reading. The results were analyzed as Lineweaver–Burk plots using Microsoft Office Excel 2013 [15].

### 3.4. Molecular Docking

The docking study was used to discover the binding modes of the most active compound, **4n**, to the BChE active site. The structure of *Homo sapiens* BChE (PDB ID: 4BDS) [32], including tacrine in the active site, was retrieved from the Protein Data Bank server (www.pdb.org). The structure of **4n** was assembled with the Schrödinger Maestro (Schrödinger, 2016) interface [37], and was then directed to the Protein Preparation Wizard protocol of the Schrödinger Suite 2016 Update 2 [38]. The LigPrep 3.8 [39] software was used to accurately prepare compound **4n** for the assignment of protonation regions at pH 8.0 ± 1.0 and the atom types. Bond orders and hydrogen atoms in the structure were fixed as required. The Glide 7.1 program [40] was used to generate the grid, and docking analyses were carried out using the single-precision docking mode (SP).

### 3.5. Cell Viability Assay and Selectivity Indexes

Healthy murine embryonic fibroblast cells (NIH3T3 cell line) were used for the cytotoxicity tests. The cell line and selected compounds were prepared for an MTT assay as reported previously [41]. The optical densities (OD) of 96 well plates were recorded at 540 nm. The percentage inhibition was determined for each concentration according to the formula below, and the IC_50_ values were calculated by applying regression analyses using Microsoft Excel 2013 [15].

$$\;\%\;Inhibition=100-\frac{\left(OD\;of\;sample\right)}{\left(OD\;of\;solvent\right)}\times 100.\;$$

### 3.6. BBB Permeability and Drug-Likeness Score (DLS)

With a specific end goal of assessing some important biological parameters for the active compounds (**4a**, **4b**, **4e**, **4i**, **4m**, **4n**, **4o**, and **4t**), BBB permeability and DLS were predicted using an online BBB Predictor and Molsoft’s chemical fingerprints mode, respectively [42,43].

## 4. Conclusions

Due to the complicated nature of AD, scientists are still trying to discover a medication to cure this disease. One possible way of overcoming this disease is through the discovery of new compounds that may be useful in its treatment. Hence, 21 new derivatives of 2-(9-acridinylamino)-2-oxoethyl-piperazinyl/piperidinyl/morpholinylcarbodithioate (**4a**–**4u**) were designed, synthesized, and screened for their inhibition potential toward ChEs. Eight derivatives (**4a**, **4b**, **4e**, **4i**, **4m**, **4n**, **4o**, and **4t**) demonstrated a specific and promising action against BChE, from which only one compound (**4n**) was able to inhibit both enzymes. BChE kinetics and molecular modeling studies were applied to investigate the binding mode of the lead compound, **4n**, to the enzyme’s active site. Moreover, the result of cytotoxic activity against NIH3T3 cells indicated that the active derivatives showed no cytotoxicity, suggesting a safety profile of these compounds. Similarly, the BBB permeability and DLS studies indicated the relative success of the most active derivatives. 

## Figures and Tables

**Table:** 

**Comp.**	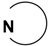	**R**	**Comp.**	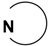	**R**
**4a**	Piperazinyl	4-(2-(dimethylamino)ethyl	**4l**	Piperazinyl	4-cyclohexyl
**4b**	Piperazinyl	4-(3-(dimethylamino)propyl	**4m**	Piperazinyl	4-benzhydryl
**4c**	Piperazinyl	4-ethyl	**4n**	Piperazinyl	4-[4-(trifluoromethyl)benzyl]
**4d**	Piperazinyl	4-(4-nitrophenyl)	**4o**	Piperazinyl	4-(4-methylbenzyl)
**4e**	Piperazinyl	4-(2-hydroxyethyl)	**4p**	Piperazinyl	4-(4-fluorophenyl)
**4f**	Piperazinyl	4-phenyl	**4q**	Piperazinyl	4-(2-furoyl)
**4g**	Piperazinyl	4-methyl	**4r**	Morpholinyl	-
**4h**	Piperazinyl	4-(4-methoxyphenyl)	**4s**	Piperidinyl	-
**4i**	Piperazinyl	4-(4-chlorophenyl)	**4t**	Piperidinyl	2-methyl
**4j**	Piperazinyl	4-(pyrimidin-2-yl)	**4u**	Piperidinyl	4-benzyl
**4k**	Piperazinyl	4-benzyl			

**Table 1:** Inhibitory activity (%) of compounds **4a**–**4u** against acetylcholinesterase (AChE) and butyrylcholinesterase (BChE) enzymes at 10^−4^ M concentration and their half maximal inhibitory concentration (IC_50_; µM) values against these enzymes.

	AChE (*Electrophorus electricus*) (%) ± SD		AChE (Human) (%) ± SD		BChE (Equine) (%) ± SD		BChE (Human) (%) ± SD	
**Compound**	10^−4^ M	IC_50_ (µM)	10^−4^ M	IC_50_ (µM)	10^−4^ M	IC_50_ (µM)	10^−4^ M	IC_50_ (µM)
**4a**	17.03 ± 0.48	-	18.15 ± 0.51	-	* 91.13 ± 2.94	0.226 ± 0.008	* 93.25 ± 2.24	0.243 ± 0.009
**4b**	32.41 ± 1.01	-	35.49 ± 1.00	-	* 82.50 ± 2.07	0.264 ± 0.012	* 84.18 ± 2.21	0.222 ± 0.010
**4c**	5.11 ± 0.09	-	10.28 ± 0.22	-	22.70 ± 0.70	-	20.55 ± 0.61	-
**4d**	31.64 ± 0.45	-	34.71 ± 0.77	-	40.19 ± 0.49	-	45.26 ± 0.60	-
**4e**	7.09 ± 0.64	-	10.13 ± 0.51	-	* 95.98 ± 2.88	1.420 ± 0.068	* 96.57 ± 2.03	1.373 ± 0.057
**4f**	4.70 ± 0.27	-	8.71 ± 0.62	-	12.80 ± 0.87	-	15.67 ± 0.79	-
**4g**	10.44 ± 0.66	-	11.30 ± 0.58	-	33.65 ± 0.89	-	35.97 ± 0.80	-
**4h**	15.23 ± 0.69	-	18.39 ± 0.57	-	12.64 ± 0.46	-	15.58 ± 0.67	-
**4i**	35.91 ± 1.57	-	33.85 ± 1.15	-	* 69.51 ± 1.16	2.038 ± 0.068	* 72.90 ± 1.08	2.097 ± 0.085
**4j**	2.88 ± 0.18	-	5.97 ± 0.22	-	27.86 ± 1.07	-	30.77 ± 1.20	-
**4k**	8.62 ± 0.22	-	7.21 ± 0.18	-	25.14 ± 1.42	-	20.65 ± 1.20	-
**4l**	3.90 ± 0.09	-	7.29 ± 0.18	-	45.88 ± 1.62	-	42.60 ± 1.53	-
**4m**	25.43 ± 1.21	-	27.38 ± 1.10	-	* 98.96 ± 3.47	0.092 ± 0.001	* 97.50 ± 2.06	0.093 ± 0.002
**4n**	* 63.21 ± 1.76	45.945 ± 1.581	* 68.39 ± 1.99	41.129 ± 1.694	* 99.03 ± 3.26	0.015 ± 0.0006	* 98.25 ± 2.63	0.014 ± 0.0005
**4o**	25.30 ± 1.01	-	30.45 ± 1.15	-	* 74.83 ± 2.17	1.071 ± 0.051	* 75.25 ± 2.08	1.069 ± 0.039
**4p**	48.40 ± 1.18	-	49.51 ± 1.50	-	48.65 ± 1.14	-	40.71 ± 1.08	-
**4q**	16.35 ± 0.39	-	20.28 ± 0.51	-	39.90 ± 1.36	-	42.48 ± 1.22	-
**4r**	37.33 ± 1.03	-	40.70 ± 1.00	-	45.30 ± 1.88	-	43.29 ± 1.17	-
**4s**	32.17 ± 1.23	-	35.20 ± 1.12	-	38.40 ± 0.74	-	34.99 ± 0.63	-
**4t**	15.94 ± 0.42	-	14.51 ± 0.44	-	* 55.00 ± 1.74	1.515 ± 0.072	* 54.26 ± 1.27	1.607 ± 0.062
**4u**	25.88 ± 0.74	-	30.71 ± 0.52	-	29.50 ± 0.48	-	31.21 ± 0.50	-
**Aminoacridine**	75.18 ± 1.15	1.752 ± 0.072	78.22 ± 0.89	1.339 ± 0.047	87.29 ± 1.07	0.233 ± 0.009	86.12 ± 1.22	0.221 ± 0.007
**Donepezil**	98.56 ± 2.87	0.0077 ± 0.0003	96.95 ± 2.14	0.0072 ± 0.0002	71.65 ± 2.16	1.683 ± 0.064	76.50 ± 2.08	1.419 ± 0.047
**Tacrine**	97.29 ± 3.24	0.147 ± 0.004	98.79 ± 2.63	0.151 ± 0.002	98.61 ± 3.71	0.0068 ± 0.0002	99.15 ± 2.07	0.0070 ± 0.0002

* Represents the selected compounds for second step due to more than 50% inhibitory potency at 10^−4^ M concentration.

**Table 2:** IC_50_ of selected derivatives against a murine fibroblast healthy cell line (NIH3T3).

Compound	IC_50_ (µM)
**4a**	26.81
**4b**	25.64
**4e**	324.90
**4i**	>1000
**4m**	214.18
**4n**	>1000
**4o**	>1000

**Table 3:** Drug-likeness score (DLS) and blood–brain barrier (BBB) permeability of the active compounds.

Comp.	DLS	BBB Permeability
**4a**	2.21	+
**4b**	2.16	+
**4e**	1.73	+
**4i**	1.80	+
**4m**	1.88	+
**4n**	1.40	+
**4o**	1.54	+
**Tacrine**	0.97	+
**Donepezil**	0.91	+

## Supplementary Materials

The following are available online, Table S1: Inhibitory Activity (%) of the compounds **4a**–**4u** against AChE and BChE., Table S2: Inhibitory activity (%) and IC_50_ values of the selected compounds against AChE and BChE.
