# Supplementary material for: Synthesis and Biological Evaluation of New Cholinesterase Inhibitors for Alzheimer’s Disease
Source: Molecules. 2018 Aug 14;23(8):2033. doi: 10.3390/molecules23082033 (PMC6222329; doi:10.3390/molecules23082033)
Supplement: Supplementary file 1 [file molecules-23-02033-s001.pdf]

molecules

MDPI

Synthesis and Biological Evaluation of New Cholinesterase Inhibitors for Alzheimer's Disease

Weiam Hussein [1,2], Begüm Nurpelin Sağlık [2,3], Serkan Levent [2,3], Büşra Korkut [4], Sinem Ilgın [4], Yusuf Özkay [2,3,*] and Zafer Asım Kaplancıklı [2]

[1] Department of Pharmaceutical Chemistry, Faculty of Pharmacy, Aden University, 6075 Aden, Yemen; white.wf.rose@gmail.com
[2] Department of Pharmaceutical Chemistry, Faculty of Pharmacy, Anadolu University, 26470 Eskişehir, Turkey; bnsaglik@anadolu.edu.tr (B.N.S.); serkanlevent@anadolu.edu.tr (S.L.); zakaplan@anadolu.edu.tr (Z.A.K.)
[3] Doping and Narcotic Compounds Analysis Laboratory, Faculty of Pharmacy, Anadolu University, 26470 Eskişehir, Turkey
[4] Department of Pharmaceutical Toxicology, Faculty of Pharmacy, Anadolu University, 26470 Eskişehir, Turkey; busrakorkut@anadolu.edu.tr (B.K.); silgin@anadolu.edu.tr (S.I.)
* Correspondence: yozkay@anadolu.edu.tr; Tel.: +90-222-335-0580 (ext. 3779)

Supplementary Materials

Table S1. Inhibitory Activity (%) of the Compounds **4a-4u** against AChE and BChE.

	AChE (Electrophorus electricus) (%)±SD		AChE (Human) (%)±SD		BChE (Equine) (%)±SD		BChE (Human) (%)±SD	
**Compound**	**10^{-3} M**	**10^{-4} M**	**10^{-3} M**	**10^{-4} M**	**10^{-3} M**	**10^{-4} M**	**10^{-3} M**	**10^{-4} M**
**4a**	60.13±1.54	17.03±0.48	62.20±1.05	18.15±0.51	99.13±3.21	*91.13±2.94	98.50±2.46	*93.25±2.24
**4b**	79.95±1.78	32.41±1.01	75.66±1.17	35.49±1.00	98.16±3.11	*82.50±2.07	97.55±2.05	*84.18±2.21
**4c**	50.73±2.21	5.11±0.09	51.25±1.45	10.28±0.22	45.18±0.94	22.70±0.70	50.23±0.88	20.55±0.61
**4d**	55.62±1.14	31.64±0.45	50.46±1.09	34.71±0.77	77.75±1.79	40.19±0.49	80.56±1.51	45.26±0.60
**4e**	48.67±1.51	7.09±0.64	50.22±1.12	10.13±0.51	97.00±3.16	*95.98±2.88	98.95±2.10	*96.57±2.03
**4f**	77.65±2.42	4.70±0.27	74.28±1.04	8.71±0.62	77.60±1.60	12.80±0.87	75.23±1.25	15.67±0.79
**4g**	26.90±0.83	10.44±0.66	30.85±0.65	11.30±0.58	78.11±2.24	33.65±0.89	80.29±2.40	35.97±0.80
**4h**	30.87±1.01	15.23±0.69	35.77±1.62	18.39±0.57	84.02±2.35	12.64±0.46	80.88±2.05	15.58±0.67
**4i**	86.41±2.91	35.91±1.57	89.20±1.25	33.85±1.15	99.37±3.41	*69.51±1.16	98.22±2.14	*72.90±1.08
**4j**	69.08±1.67	2.88±0.18	66.71±1.06	5.97±0.22	92.87±2.84	27.86±1.07	90.51±2.18	30.77±1.20
**4k**	58.74±1.69	8.62±0.22	59.17±1.11	7.21±0.18	74.62±2.75	25.14±1.42	70.98±2.39	20.65±1.20
**4l**	56.11±2.46	3.90±0.09	58.33±1.39	7.29±0.18	84.88±3.34	45.88±1.62	85.71±2.40	42.60±1.53
**4m**	71.23±2.15	25.43±1.21	75.18±1.21	27.38±1.10	99.65±3.86	*98.96±3.47	98.18±2.71	*97.50±2.06
**4n**	80.99±2.65	*63.21±1.76	83.27±1.78	*68.39±1.99	99.49±2.97	*99.03±3.26	99.33±2.10	*98.25±2.63
**4o**	94.97±3.61	25.30±1.01	90.29±2.18	30.45±1.15	98.64±3.86	*74.83±2.17	99.25±2.63	*75.25±2.08
**4p**	98.66±2.98	48.40±1.18	96.90±2.17	49.51±1.50	93.27±3.07	48.65±1.14	92.55±2.51	40.71±1.08
**4q**	68.60±1.57	16.35±0.39	70.29±1.18	20.28±0.51	92.00±3.44	39.90±1.36	95.29±2.16	42.48±1.22
**4r**	68.20±1.69	37.33±1.03	64.93±1.21	40.70±1.00	91.26±3.42	45.30±1.88	94.18±2.62	43.29±1.17
**4s**	44.51±0.96	32.17±1.23	49.05±0.88	35.20±1.12	86.72±3.07	38.40±0.74	84.27±2.16	34.99±0.63
**4t**	81.22±2.77	15.94±0.42	85.44±1.72	14.51±0.44	96.06±3.61	*55.00±1.74	97.28±3.00	*54.26±1.27
**4u**	69.30±1.28	25.88±0.74	65.77±1.17	30.71±0.52	32.20±0.59	29.50±0.48	38.33±0.87	31.21±0.50
**Aminoacridine**	85.22±1.08	75.18±1.15	87.19±0.97	78.22±0.89	91.25±1.20	87.29±1.07	90.36±1.04	86.12±1.22
**Donepezil**	99.48±3.29	98.56±2.87	98.50±2.46	96.95±2.14	82.24±2.91	71.65±2.16	85.17±2.01	76.50±2.08
**Tacrine**	99.16± 2.49	97.29± 3.24	99.85± 2.21	98.79± 2.63	99.27± 2.86	98.61±3.71	99.82±2.23	99.15± 2.07

*represents the selected compounds due to more than 50% inhibitory potency at 10^{-4} M concentration.

Table S2. Inhibitory activity (%) and IC_{50} values of the selected compounds against AChE and BChE.

Compound	10^{-5} M	10^{-6} M	10^{-7} M	10^{-8} M	10^{-9} M	IC_{50} (μM)
**AChE (Electrophorus electricus) (%)±SD**						
**4n**	22.41±0.80	14.56±0.76	11.31±0.61	8.43 ±0.38	6.91±0.27	45.945 ±1.581
**Aminoacridine**	58.97±1.05	41.71±0.97	31.56±0.82	20.40±0.55	15.98±0.48	1.752±0.072
**Donepezil**	95.30±1.64	92.15± 1.88	81.36± 1.41	41.78± 0.84	25.62± 0.58	0.0077 ±0.0003
**Tacrine**	95.95 ±2.16	75.05 ±2.21	36.64 ±1.94	20.40 ±0.67	17.05 ±0.44	0.147 ±0.004
**AChE (Human) (%)±SD**						
**4n**	18.46±0.54	10.52±0.37	9.18±0.22	5.51 ±0.17	3.00±0.12	41.129 ±1.694
**Aminoacridine**	55.30±0.82	44.29±0.74	35.77±0.67	21.58±0.42	16.74±0.37	1.339±0.047
**Donepezil**	96.88±1.25	93.00± 1.12	80.47± 1.07	45.19± 0.77	23.63± 0.49	0.0072 ±0.0002
**Tacrine**	93.36 ±1.35	74.58 ±1.52	39.22 ±0.97	21.19±0.52	16.98 ±0.48	0.151 ±0.002
**BChE (Equine) (%)±SD**						
**4a**	84.80±2.76	52.10±1.04	39.90±0.81	28.24±0.54	19.69±0.99	0.226 ±0.008
**4b**	70.30 ±1.80	46.10±0.74	41.80±0.81	33.04±0.78	26.35±0.77	0.264 ±0.012
**4e**	57.53±1.89	29.52±1.07	24.26±0.54	18.89±0.84	10.77±0.58	1.420 ±0.068
**4i**	44.32±0.79	38.72±0.82	33.65±0.45	26.52±0.80	25.73±0.70	2.038 ±0.068
**4m**	90.78±3.92	80.30±2.56	40.34±0.61	30.24±0.70	18.98±0.20	0.092 ±0.001
**4n**	92.51±2.86	81.28±1.25	49.33±0.58	43.73±1.11	39.25±0.22	0.015 ±0.0006
**4o**	46.33±0.91	42.99±0.97	35.48±0.67	25.36±0.49	16.54±0.56	1.071 ±0.051
**4t**	49.04±0.68	41.33±0.41	35.75±0.53	30.66±0.59	28.50±0.66	1.515 ±0.051
**Aminoacridine**	71.55±1.02	56.26±0.97	42.75±0.63	30.11±0.48	24.88±0.50	0.233±0.009
**Donepezil**	59.24±1.54	54.27±1.16	38.14±0.89	15.29±0.61	9.23 ±0.08	1.683 ±0.064
**Tacrine**	94.27±3.19	91.68±2.82	84.27±2.12	46.27 ±1.09	22.49±0.70	0.0068 ±0.0002
**BChE (Human) (%)±SD**						
**4a**	86.20±1.46	55.00±1.11	36.21±0.97	30.20±0.68	15.79±0.81	0.243 ±0.009
**4b**	72.99 ±1.35	45.26±0.85	40.87±0.67	35.28±0.53	28.74±0.50	0.222 ±0.010
**4e**	55.00±1.20	30.62±1.28	26.54±0.74	17.89±0.53	8.72±0.30	1.373 ±0.057
**4i**	42.99±1.02	35.00±0.87	30.49±0.56	21.77±0.40	20.69±0.38	2.097 ±0.085
**4m**	92.54±2.45	81.25±2.08	44.61±0.79	29.11±0.63	17.00±0.33	0.093 ±0.002
**4n**	93.67±2.45	83.22±1.07	50.48±0.69	43.59±0.84	37.19±0.69	0.014 ±0.0005
**4o**	48.19±1.12	41.37±0.95	37.90±0.71	24.71±0.55	20.80±0.48	1.069 ±0.039
**4t**	48.19±1.08	38.35±0.88	36.47±0.60	32.40±0.57	29.77±0.41	1.607 ±0.062
**Aminoacridine**	74.27±1.08	53.28±0.99	46.82±0.70	31.92±0.62	22.70±0.48	0.221±0.007
**Donepezil**	60.18±1.37	52.49±1.05	32.70±0.95	18.55±0.74	10.44 ±0.33	1.419 ±0.047
**Tacrine**	95.23±2.25	93.47±2.18	80.79±1.04	44.08 ±0.97	25.12±0.52	0.0070 ±0.0002
